# A Novel Signaling Pathway Required for Arabidopsis Endodermal Root Organization Shapes the Rhizosphere Microbiome

**DOI:** 10.1093/pcp/pcaa170

**Published:** 2021-01-22

**Authors:** Julius Durr, Guilhem Reyt, Stijn Spaepen, Sally Hilton, Cathal Meehan, Wu Qi, Takehiro Kamiya, Paulina Flis, Hugh G Dickinson, Attila Feher, Umashankar Shivshankar, Shruti Pavagadhi, Sanjay Swarup, David Salt, Gary D Bending, Jose Gutierrez-Marcos

**Affiliations:** 1 School of Life Sciences, University of Warwick, Coventry CV4 7AL, UK; 2 Division of Plant and Crop Sciences, Future Food Beacon of Excellence & School of Biosciences, University of Nottingham, Nottingham LE12 5RD, UK; 3 Department of Plant Microbe Interactions & Cluster of Excellence on Plant Sciences (CEPLAS), Max Planck Institute for Plant Breeding Research, Carl-von-Linn�-Weg 10, K�ln 50829, Germany; 4 Centre for Microbial and Plant Genetics, Leuven Institute for Beer Research, University of Leuven, Gaston Geenslaan 1 B-3001, Belgium; 5 Graduate School of Agricultural and Life Sciences, University of Tokyo, Tokyo 113-8657, Japan; 6 Department of Plant Sciences, University of Oxford, Oxford OX1 3RB, UK; 7 Institute of Plant Biology, Biological Research Centre of the Hungarian Academy of Sciences, Temesv�ri krt. 62, Szeged H-6726, Hungary; 8 Department of Biological Sciences, National University of Singapore, 14 Science Drive 4, 117543, Singapore

**Keywords:** Endodermis, Metabolome, Microbiome, Phosphorylation, Signaling, Suberin

## Abstract

The Casparian strip (CS) constitutes a physical diffusion barrier to water and nutrients in plant roots, which is formed by the polar deposition of lignin polymer in the endodermis tissue. The precise pattern of lignin deposition is determined by the scaffolding activity of membrane-bound Casparian Strip domain proteins (CASPs), but little is known of the mechanism(s) directing this process. Here, we demonstrate that Endodermis-specific Receptor-like Kinase 1 (ERK1) and, to a lesser extent, ROP Binding Kinase1 (RBK1) are also involved in regulating CS formation, with the former playing an essential role in lignin deposition as well as in the localization of CASP1. We show that ERK1 is localized to the cytoplasm and nucleus of the endodermis and that together with the circadian clock regulator, Time for Coffee (TIC), forms part of a novel signaling pathway necessary for correct CS organization and suberization of the endodermis, with their single or combined loss of function resulting in altered root microbiome composition. In addition, we found that other mutants displaying defects in suberin deposition at the CS also display altered root exudates and microbiome composition. Thus, our work reveals a complex network of signaling factors operating within the root endodermis that establish both the CS diffusion barrier and influence the microbial composition of the rhizosphere.

## Introduction

Plant roots are specialized structures essential for plant growth and survival. Roots control the selective uptake of nutrients and water from the soil, and also prevent the passive diffusion and entry of pathogens and toxins (Hawes et al. 1998, Parniske 2008), while attracting beneficial microorganisms through the secretion of certain compounds into the soil (Sasse et al. 2018). This selectivity is determined by the root architecture and its ability to confer a barrier between the vascular cylinder and the outer cell layers, primarily cortex and epidermis, which are connected to the soil via the apoplast to control the uptake and the exudation of compounds. The apoplastic barrier provided by the Casparian strip (CS) is contained within the endodermis; the innermost cortical cell layer surrounding the vasculature (Bonnett 1968, Nagahashi et al. 1974). These specialized groups of cells possess highly localized ring-like lignin deposits in the radial and transverse cell walls surrounding the endodermis (Naseer et al. 2012), which seals the cell wall and breaks the apoplastic connection between the vasculature and the outer cell layer.

The endodermal cell wall undergoes further modification by the incorporation of suberin—a hydrophobic polymer composed of long chain fatty acids and glycerol embedded with waxes (Baxter et al. 2009), which constitutes a second barrier. The suberin lamellae ensure that, in fully differentiated endodermal cells, nutrients can only enter or leave the vasculature through specialized transmembrane carriers or special unsuberized passage cells (Andersen et al. 2018, Doblas et al. 2017a, Graca and Santos 2007). The extent of suberin deposition is dynamic and mediated by abscisic acid and ethylene in response to nutrient stresses (Barberon et al. 2016) or in the case of a defective CS (Wang et al. 2019). Further, suberization of the CS shows high plasticity in contrast to lignification of the CS.

In Arabidopsis, the formation of the CS apoplastic barrier is regulated by the transcription factor MYB36, which controls the expression of major genes involved in CS formation during the initial stages of endodermis differentiation (Kamiya et al. 2015). The first major MYB36-dependent initiation step that gives rise to CS formation is the polar localization of five redundant CASPARIAN STRIP DOMAIN PROTEINs (CASP1-5) at the site of CS initiation (Roppolo et al. 2011). The CASPs form a platform to recruit proteins involved in polar lignin deposition, such as endodermis-specific peroxidase PEROXIDASE64 and NADPH oxidase F (RbohF) (Lee et al. 2013). Several mutants have been identified that affect CASP localization to the endodermis. The two receptor-like kinase mutants *schengen 1* (*sgn1)* and *schengen 3* (*sgn3*), and the dirigent domain-containing protein mutant *enhanced suberin 1* (*esb1*) exhibit only a discontinuous CS, while *CASP* expression and lignification of the CS domain remain unaffected (Alassimone et al. 2016, Hosmani et al. 2013, Pfister et al. 2014). By contrast, other CS mutants, such as *lord of the rings 1* and *2* (*lotr1* and *lotr2*), display relatively strong ectopic localization of CASP proteins outside of the CS domain and irregular lignification (Kalmbach et al. 2017, Li et al. 2017).

Despite these findings, the precise mechanisms underlying formation of the endodermal barriers, and in particular CS lignification, remain poorly understood. To further understand these events, we performed a reverse genetic screen to identify novel signaling components involved in endodermis development. Here, we report two cytoplasmic receptor-like kinases that specifically accumulate in the root endodermis and are involved in the formation of the CS apoplastic barrier. We show that these kinases facilitate the polar localization of CASP1 and the polar deposition of lignin at the CS domain. In addition, we provide evidence that the circadian clock regulator protein, TIME FOR COFFEE (TIC), is a downstream target of these kinases and is also involved in CS organization. Finally, we also show that the correct deposition of lignin and suberin in the endodermis is critical for the release of root exudates and for selective recruitment of beneficial microbes to roots.

## Results

### Identification of two cytoplasmic receptor-like kinases implicated in the formation of the endodermal barriers

To identify novel components of the signaling pathway implicated in the formation of the CS, we carried out an in silico analysis of receptor-like kinases (RLK) that are under the control of MYB36—a transcription factor implicated in endodermis gene expression and CS formation (Kamiya et al. 2015). This analysis identified a candidate, RLK (At5g65530/ARLCK VI_A3), hereafter named ENDODERMIS RECEPTOR KINASE1 (ERK1) (Supplementary Fig. S1A). We generated ERK1-GFP lines, which revealed ERK1 accumulation in the cytoplasm and nucleus of endodermal root cells only; its expression was first detected in the late elongation zone and was highest in the region where the first xylem bundles are formed (Fig.�1A). Using chromatin immunoprecipitation, we also determined that MYB36 directly targets a discrete region of the ERK1 promoter (Fig.�1B). We investigated the expression of the closest homologue of ERK1, ROP binding kinase1 (RBK1), and found that it was largely restricted to the root endodermis (Supplementary Fig. S1B). Both ERK1 and RBK1 lack the extracellular and transmembrane domains usually present in other receptor-like kinases (Afzal et al. 2008) but have a conserved serine/threonine kinase domain that is activated by phosphorylation through small monomeric G proteins of the plant-specific Rho family (RAC/ROP) (Huesmann et al. 2012). While genetic analyses of ERK1 and RBK1 have previously revealed their roles in trichome branching and in the control of basal resistance to powdery mildew (Huesmann et al. 2012, Reiner et al. 2015), and in auxin-responsive cell expansion (Enders et al. 2017) and pathogen response (Molendijk et al. 2008), respectively, their roles in endodermal function are unknown. We therefore isolated independent T-DNA insertion lines for both genes (Supplementary Fig. S1C) and assessed mutants for defects in apoplastic barrier diffusion determined by leakage analysis of the apoplastic tracer propidium iodide (PI) into the stele of roots (Reiner et al. 2015). We found that all ERK1 mutant lines (*erk1-1*/SALK_148741, *erk1-2*/SALK_010841 and *erk1-3*/SALK_060966) showed defects in the formation of the apoplastic barrier in the endodermis (Fig.�1C, D). We were able to restore the apoplastic barrier defects observed in *erk1-3* by complementation with a wild-type *ERK1* genomic fragment (Supplementary Fig. S1D), suggesting that the CS defects observed are due to the loss of *ERK1* function. On the other hand, we found that RBK1 mutants (*rbk1-1*/SALK_043441) did not display any obvious defects (Fig.�1C, D). Notably, *erk1-3*/*rbk1-1* mutants showed a significantly stronger defective apoplastic barrier phenotype than either of the individual single mutants, as measured by PI penetration (Fig.�1C, D), suggesting that *rbk1-1* has an additive effect in the absence of *ERK1.*

**Fig. 1:**
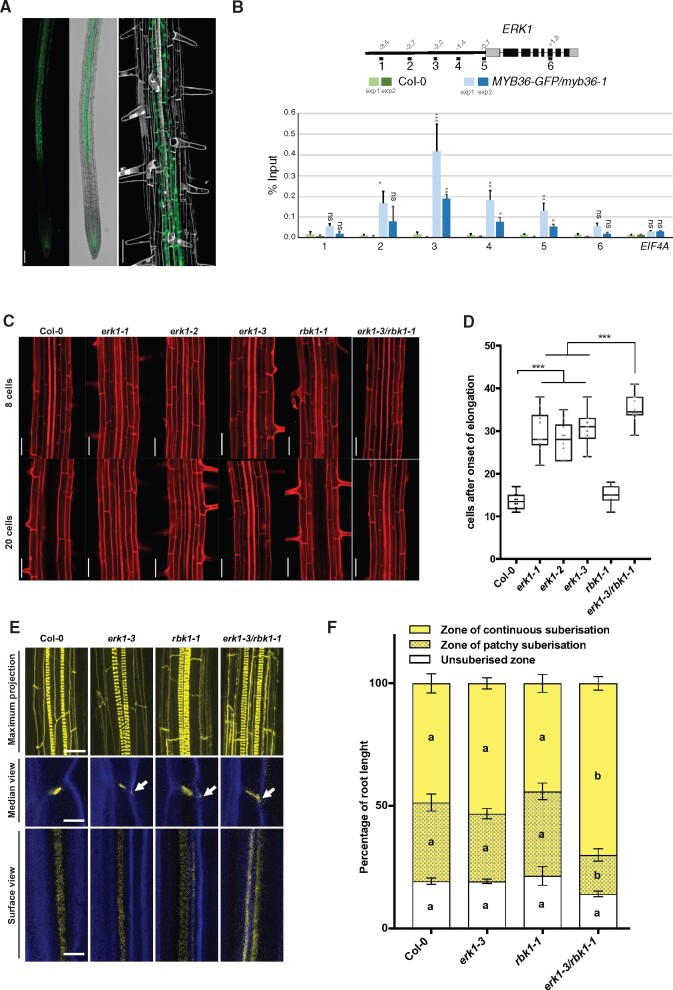
Loss of CS integrity and disruption of the apoplastic barrier in *erk1* and *rbk1* mutants. (A) Confocal microscopy images of roots expressing ERK1–GFP. Cell walls stained with propidium iodide (grey). Bar = 200 �M. (B) Chromatin immunoprecipitation shows that MYB36 binds the promoter of *ERK1* (*n* = 3). EIF4A was used as control. ChIP-qPCR data are shown as means � S.D from 3 technical replicates. Student's *t*-test, *P <0.1, **P<0.05, ***P<0.001 and n.s. indicate no significance. (C) Lack of endodermal diffusion barrier in *erk1* and *rbk1* mutants visualized by presence of propidium iodide (red) in stele. Bar = 50 �M. (D) Quantification of PI penetration into the stele quantified as number of endodermal cells from the first fully expanded cell (*n* = 10). Differences between groups were determined by paired *t*-test, ***P < 0.001. (E) Three-dimensional maximum projections of CS autofluorescence. Spiral structures in the center of the root are xylem (top). Longitudinal section of lignin deposition sites (bottom). Cleared roots were stained with basic fuchsin (yellow; lignin) and Calcofluor White (blue; cellulose). Although both of these dyes stain cell walls, basic fuchsin primarily interacts with lignin and Calcofluor White with cellulose. White arrows indicate the dispersed deposition of lignin at the CS. (F) Quantitative analysis of suberin accumulation. Suberin was stained with fluorol yellow 088. The endodermal cell suberin was counted from the onset of elongation to the junction (base) between root and hypocotyl (*n* = 6). Individual letter shows significant differences using Mann–Whitney test between the same zones (*P* < 0.01).

To determine of the cause of the apoplastic barrier defects found in *erk1* mutants, we investigated the cell wall deposition in CS by transmission electron microscopy (TEM). We found that the cell wall deposition at the CS was enlarged in *erk1-3* mutants compared to wild-type (Supplementary Fig. S1E). To further investigate the endodermis defects found in *erk1* mutants, we then examined the pattern of lignin and cellulose deposition in the CS using dyes and confocal microscopy. Compared to wild-type roots, the single *erk1-3* and *rbk1-1* mutants showed a slight accumulation of ectopic lignin in the corners of endodermal cells, though ectopic lignin was more apparent in *erk1-3*/*rbk1-1* mutants (Fig.�1E). Given that precocious and ectopic deposition of suberin has also been reported in the endodermis of other mutants harboring similar disruptions to the CS, such as *esb1-1*, *casp1-1/casp3-1*, *myb36-2* and *lcs2-1* (Hosmani et al. 2013, Kamiya et al. 2015, Li et al. 2017,Roppolo et al. 2011), we also assessed suberin content in our mutants. We found that suberization in single *erk1-3* and *rbk1-1* mutants was normal, while *erk1-3*/*rbk1-1* roots showed premature suberization and resulted in an enlarged deposition of continuous suberin in early differentiated endodermal cells (Fig.�1F). Collectively, these data suggest that both ERK1 and, to a lesser extent, RBK1 are necessary for the correct deposition of lignin and suberin in the root endodermis.

### Downstream targets of ERK1 are required for CS barrier formation

It has been reported that ERK1 is capable of phosphorylating proteins in vitro (Reiner et al. 2015). Therefore, to identify the downstream targets of ERK1 in the endodermis, we performed a mass spectrometry-based quantitative phosphoproteomics analysis using roots from wild-type (Col-0) and *erk1-3* plants. We identified ∼100 proteins displaying over 1.5-fold significant change in phosphorylation (Supplementary Table S1). To reveal the potential targets and pathways affected, we performed a Gene Ontology (GO) analysis and found sequences associated with the terms ‘response to abscisic acid’, ‘establishment of localization’ and ‘translation’ to be significantly enriched (*P* < 0.001) (Supplementary Fig. S2A). One of these proteins was RBOHD, which has been recently found to be part of ROS production and lignification in the CS (Fujita et al. 2020). Notably, TIME FOR COFFEE (TIC) and TOM-LIKE6 (TOL6) were found to be differentially phosphorylated in *erk1-3* (Supplementary Table S1 and Fig. S2B). Although these proteins have been implicated in different aspects of plant development, they have not been linked to CS formation. We assessed the permeability of the apoplastic tracer PI into the stele in the respective mutant backgrounds and observed a large delay in the formation of the PI block in two independent *tic* mutants, which was significantly greater than in the *erk1-3* mutant allele (Fig.�2A). By contrast, we did not see any increased permeability in *tol6-1* or in combinations of other *TOL* mutants (Fig.�2A and Supplementary Fig. S3A). To evaluate if the apoplastic barrier defect observed in *tic* mutants is a generic circadian clock defect, we analyzed three other circadian clock mutants (*elf3-4*, *cca1-1* and *elf4-7)* and overexpression lines (ELF3-OX and CCA1-OX); however, none of these lines showed any differences in apoplastic permeability with respect to the wild-type (Supplementary Fig. S3B). We then inspected the deposition of lignin in the endodermis of *tic* and *tol* mutants and found that both *tic-2* and the quintuple *tolQ* lines showed strong ectopic deposition of lignin in the lateral margins of endodermal cells (Fig.�2B). Intriguingly, we found that only *tic* mutants showed precocious accumulation of suberin in the endodermis (Fig.�2C). Collectively, these results suggest that ERK1 and TIC act in the same pathway and are responsible for the organization of the CS. We then assessed PI penetration, lignin deposition and suberin accumulation in mutants of *erk1-3* in combination with *tic-2* and general CS mutants *myb36-2, sgn3-3, esb1-1* and *casp1-3/casp3-1*. We did not find significant additive effects for PI penetration and lignin deposition in double *erk1-3/tic2* mutants compared with single *erk1-3* mutants, nor in any of the other double mutants compared with the single mutants, except for *erk1-3/sgn3-3*, which seemed to contain slightly less lignin than *sgn3-3* (Supplementary Fig. S4A, B). When assessing suberin accumulation, again, no major differences were observed; however, triple and double mutants of *erk1-3* with *casp1-3/casp3-1* and *myb36-2*, respectively, appeared to have slightly less suberin (Supplementary Fig. S4C).

**Fig. 2:**
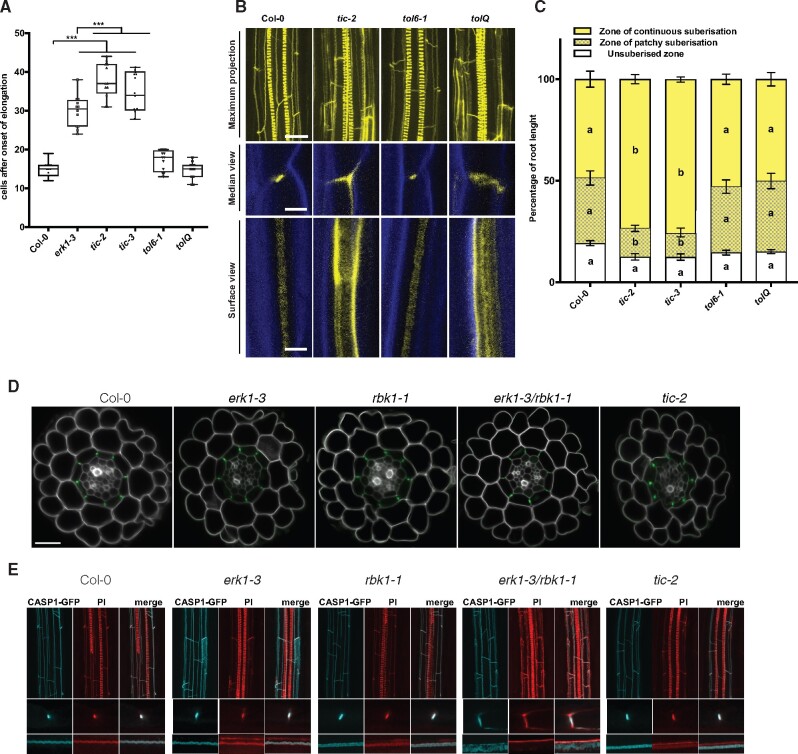
*TIC* and *TOL* are downstream targets of the ERK1 signaling pathway implicated in Casparian Strip formation. (A) Quantification of PI penetration into the stele quantified as number of endodermal cells from the first fully expanded cell (*n* = 10). Differences between groups were determined by paired *t*-test, ***P < 0.001. (B) Three-dimensional maximum projections of CS autofluorescence. Spiral structures in the center of the root are xylem (top). Longitudinal section of lignin deposition sites (bottom). Cleared roots were stained with basic fuchsin (yellow; lignin) and Calcofluor White (blue; cellulose). (C) Quantitative analysis of suberin accumulation. Suberin was stained with fluorol yellow 088. The endodermal cell with suberin was counted from the onset of elongation to the junction (base) between root and hypocotyl (*n* = 6). Individual letter shows significant differences using Mann–Whitney test between the same zones (*P* < 0.01). (D) Confocal microscopy images of cross-section from roots expressing CASP1–GFP. Cell walls stained with PI (grey). Bar = 20 �M. (E) Three-dimensional maximum projections of the mature endodermis expressing CASP1–GFP and stained with basic fuchsin (lignin, red) (top) on cleared roots. Median and surface view of mature endodermal cells expressing CASP1–GFP and stained with basic fuchsin (bottom). White arrows indicate dispersed localization of CASP1-GFP and lignin.

Two recently identified stele-expressed peptides, CASPARIAN STRIP INTEGRITY FACTORS 1 & 2 (CIF1 & 2), which bind to the SGN3 receptor and promote CS formation, have been found to enhance suberin deposition in wild-type plants and induce CS mislocalization as well as overlignification (Doblas et al. 2017b, Nakayama et al. 2017). To determine whether ERK1 and RBK1 kinases are linked with the CIF/SGN3 signaling pathway, we assessed lignin deposition, CASP1-GFP expression and suberin accumulation in response to exogenous application of the CIF peptide. Our data show that CIF2 induced suberinization, ectopic polymerization and mislocalization of CASP1-GFP similar to wild-type, suggesting that the role of ERK1/RBK1 in suberin accumulation may be part of an additional compensatory pathway independent of the SGN3/CIF pathway (Supplementary Fig. S5).

The ectopic deposition of lignin in *erk1-3/rbk1-1* and *tic-2* mutants raises the possibility that the CS machinery is mislocalized in these mutants. To evaluate this hypothesis, we assessed the localization of the CASP1-GFP reporter in different mutant backgrounds. We did not observe any major defects in the cellular organization of root optical cross-sections in any of the mutants tested (Fig.�2B). However, in transverse root cross-sections, we noticed that in *erk1-3*/*rbk1-1* mutants, localization of CASP1-GFP was not restricted to the CS, but displayed a broader distribution (Fig.�2D, E). In addition, we found in *erk1-3*/*rbk1-1* mutants that the deposition of lignin was not limited to the CS domain, but instead it was broadly distributed in the endodermal cell wall (Fig.�2D, E). Taken together, these data suggest that ERK1 and RBK1 affect the polar localization of CASP proteins and the deposition of lignin in the endodermis.

### The endodermal defects in *erk1erk1-3* and *tic-2* lead to ionomic changes

Root suberization and the CS have been shown to play a role in environmental adaptation by acting as a physical barrier to prevent unfavorable inward and outward leakage of ions between the xylem and the soil (Barberon et al. 2016). In previous studies, it was shown that several CS-defective mutants exhibit changes in concentrations of multiple elements. Therefore, we compared the ionomic profiles of *erk1-3, rbk1-*1 and *tic-2* mutants with other mutants known to be defective in CS function. A principal-component analysis of elemental concentrations in the leaves showed that the ionomic profile of the *erk1-3* and *rbk1-*1 mutants was similar to wild-type, whereas the *erk1-3/rbk1-*1 double mutant was more similar to *esb1-1* but distinct to either *myb36-2* or *sgn3-3* mutants (Supplementary Fig. S6A). Unlike *sgn3-3, esb1-1* and *myb36-2* have enhanced lignification and suberization (Hosmani et al. 2013). Further analysis of the ionomic data revealed that *erk1-3/rbk1-*1 mutants accumulated higher levels of iron in their roots compared with wild-type plants (Fig.�3A). Because iron is more soluble in acidic soils than in neutral soils, we reasoned that this response may reflect an adaptation to ensure the growth and survival of plants under unfavorable mineral conditions. We therefore tested the effect of iron on ERK1-GFP accumulation and found that ERK1-GFP expression in the endodermis increased in response to excessive iron in a pH-dependent manner (Fig.�3B, C). Because a previous genetic screen for iron homeostasis mutants identified TIC as a regulator of FERRITIN1 (Duc et al. 2009), we tested the growth response of these mutants under high iron growth conditions. We found that both *erk1-3* and *tic-2* displayed high sensitivity to elevated iron conditions (Fig.�3D, E). Moreover, we also found that *sgn3-3*, but not in *myb36-2*, was highly sensitivity to high iron growth conditions (Supplementary Fig. S6B). Collectively, these results suggest that these mutants are compromised in their responses to fluctuating iron levels most likely due to the inward and outward leakage resulting from their defective endodermal barriers.

**Fig. 3:**
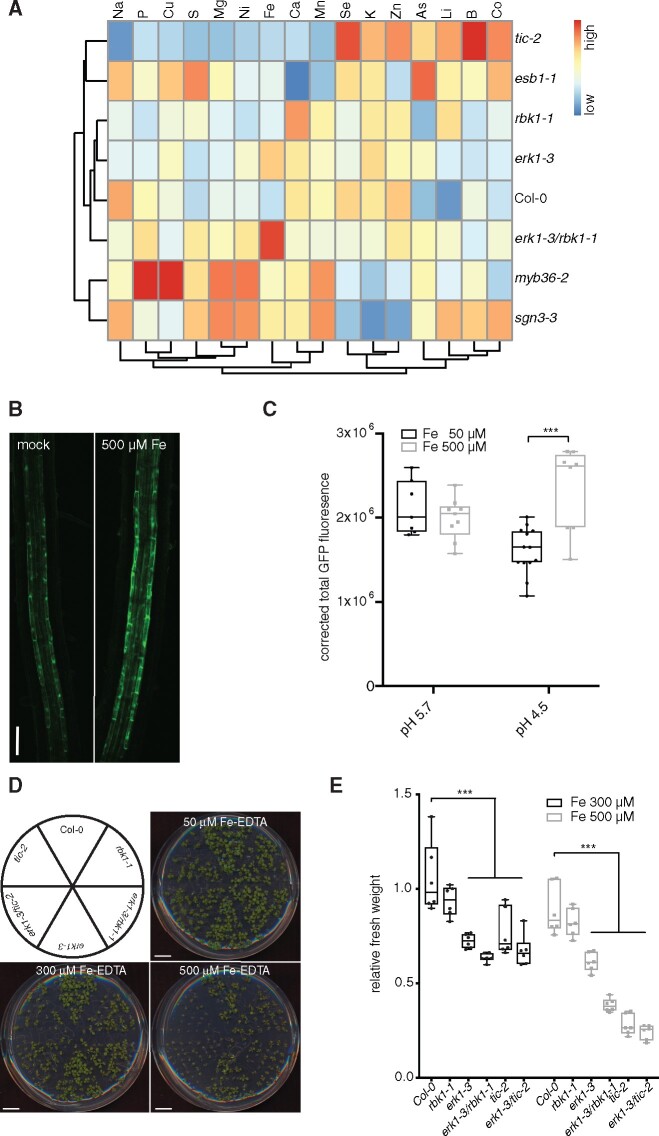
*ERK1* and *TIC* mutants display notable ionomic changes and sensitivity to excess iron. (A) Heat-map showing differences in ion accumulation in shoots from wild-type and CS mutants (*n* = 15). (B) Induction of ERK1-GFP expression in root endodermis by elevated iron-EDTA. (C) Quantification of ERK1-GFP expression in endodermis by iron-EDTA at different pH (*n* = 10). Differences between groups were determined by paired *t*-test, ***P < 0.001. (D) Sensitivity of plants grown in vitro with different iron concentrations. (E) Relative mean fresh weight of plants grown in media with elevated iron-EDTA. (*n* = 6). Differences between groups were determined by paired *t*-test, ***P < 0.001.

### The root endodermal barrier influences the structure of the root microbial community

It has previously been shown that altered exudation from the plant root causes changes in the composition of the rhizosphere microbiome (Sasse et al. 2018). We therefore tested whether the unregulated leakage of components into and out of *erk1-3* and *tic-2* mutants might also affect their respective root microbiomes. To this end, we grew wild-type and single mutant plants in natural soils, and we analyzed the bacterial communities present in the root rhizosphere 4 weeks later. We found clear differences between the genotypes present in each subpopulation; the communities associated with *erk1-3* and *tic-2* mutants were most similar but differed significantly from wild-type plants (Fig.�4A). Analysis of similarities (ANOSIM) revealed significant variations in the microbial communities from roots of wild-type plants and *erk1-3* (ANOSIM, r = 0.921, *P* = 0.001) or *tic-2* plants (ANOSIM, r = 0.922, *P* = 0.001), whereas no significant differences were observed between the *erk1-3* and *tic-2* microbiomes (ANOSIM, r=−0.019, *P* = 0.558) (Fig.�4A).

**Fig. 4:**
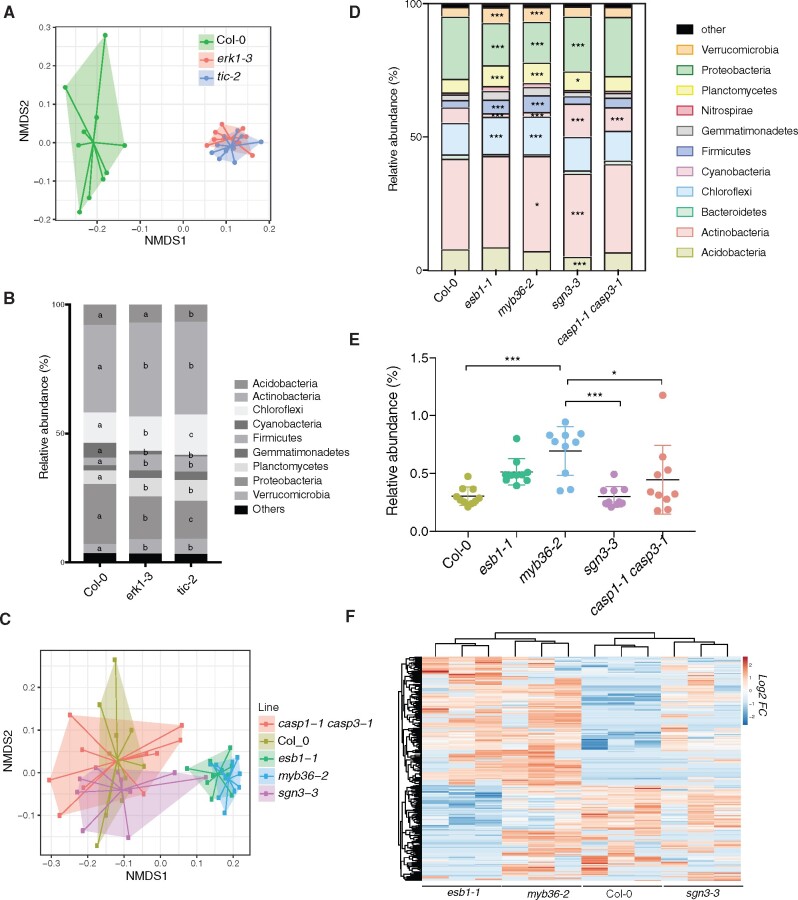
Differences in rhizosphere microbial community present in *erk1-3*, *tic-2* and other *CS* mutants. (A) Principal component analysis (PCA) of Bray-Curtis distances of bacterial communities present in roots of Col-0, *erk1-3* and *tic-2* plants grown in natural soils (*n* = 10). (B) Distribution of soil bacteria in roots of Col-0, *erk1-3* and *tic-2* plants (*n* = 10). Individual letter shows significant differences using Mann–Whitney test between the same zones (*P* < 0.01). (C) PCA of Bray-Curtis distances of bacterial communities present in roots of Col-0, *casp1-1; casp3-1, esb1-1, myb36-2* and *sgn3-3* plants grown in natural soils (*n* = 10). Asterisks shows significant differences using Mann–Whitney test between the same zones (*P* < 0.01). (D) Distribution of soil bacteria in roots of Col-0, *casp1-1; casp3-1, esb1-1, myb36-2* and *sgn3-3* plants. Asterisks shows significant differences using Mann–Whitney test between the same zones (*P < 0.01, ***P < 0.0001). (E) Colonization assay with *Bacillus amilloliquefaciens* in roots of Col-0, *casp1-1; casp3-1, esb1-1, myb36-2* and * sgn3-3* plants. (F) Unsupervised hierarchical cluster heatmap analysis of metabolites exudated from roots of Col-0, *esb1-1, myb36-2 and sgn3-3* plants. FC, fold change.

We next carefully examined the different phyla of root-associated microbes and identified several groups that differed in abundance between the *erk1-3* and *tic-2*-associated populations. Notably, Chloroflexi and Proteobacteria populations differed significantly among the three genotypes tested (Fig.�4B). To independently validate these results, we inoculated seeds with a synthetic bacterial community (SynCom) isolated from an Arabidopsis root rhizosphere (Bai et al. 2015) and determined the microbial communities present in mature roots and leaves. We found differences between the microbiomes present in leaves and roots of wild-type and the two mutants (Supplementary Fig. S7A). Most of the differences were associated with an abundance of *Xanthomonadaceae* (*Proteobacteria*) and *Flavobacteriaceae* (*Bacteroidetes*) (Supplementary Fig. S7B). To determine if the differences observed in the microbiome may be linked to defects in the apoplastic barrier, we analyzed the bacterial communities present in the root rhizosphere of *casp1*/3, *esb1*, *sgn3* and *myb36* mutants. We found that the bacterial communities of *myb36* and *esb1*, both defective in lignin and suberin deposition in CS, were notably different from *sgn3*-3 or wild-type plants (Fig.�4C). Further analysis revealed that Chloroflexi, Firmicutes, Planctomycetes, Proteobacteria and Verrucomicrobiota populations differed significantly among *myb36* and *esb1* and the other genotypes tested (Fig.�4D). To independently validate these findings, we inoculated roots of these mutants with a GFP-tagged strain of *Bacillus amiloliquefaciens*, a beneficial endophyte that promotes seedling growth. We found that the abundance and level of colonization of this endophyte was greater in *esb1* and *myb36* than in wild type and other CS mutants (Fig.�4E). To test if differences in root colonization may be linked to differences in the production of root metabolites, we performed a metabolome analysis of root exudates in mutants defective on endodermis function. We found that *esb1-1* and *myb36-2* exhibited notable similarities with the metabolites exudated (72.2%, *n* = 372), which differed in abundance to the exudates found in wild-type and *sgn3-3* plants (Fig.�4F). These results suggest that the root colonization of microbes is strongly influenced by the secretion of specific root metabolites determined by the lignification and suberization status of the endodermis.

## Discussion

The polarized deposition of cell wall material is crucial for the function and development of many root cell types (Roppolo and Geldner 2012). One of the most-studied examples of polar cell wall deposition is the root endodermis and its ring-like CS that prevents the inward and outward leakage of metabolites from the vasculature (Doblas et al. 2017a). A step in the separation of the inner and outer facing plasma membrane domains is the proper localization of CASP proteins at the site of lignin deposition (Roppolo et al. 2011). Various CS-defective mutants have been described in recent years, the majority of which also show a mislocalization of CASP proteins at the site of lignification. Three different classes of CS mutants have been identified according to CASP1 expression and localization: i) those with no CASP1-GFP expression, such as in the loss-of-function *myb36-3* mutant; ii) mutants with defects in the continuity of the CASP domain, such as *sgn1*, *sgn3* and *esb1* (Alassimone et al. 2016, Hosmani et al. 2013, Pfister et al. 2014); and iii) mutants with ectopic CASP deposition, such as *lotr1/lcs2* or *lotr2*/*exo70a1* (Kalmbach et al. 2017, Li et al. 2017).

In this study, we describe mutations in the cytoplasmic receptor kinases ERK1 and RBK1, which lead to the mislocalization of CASP1-GFP and ectopic lignin deposition outside of the CS. Further, we show that double mutants lack a proper apoplastic barrier but show increased lignin and suberin, which has been attributed to a compensatory mechanism (Doblas et al. 2017a) and likely dependent on the membrane-bound and cytoplasmic kinase module SNG3/SGN1 (Alassimone et al. 2016, Pfister et al. 2014). The mechanism by which the ERK/RBK1 signaling module affects lignin deposition and suberization is currently unclear; however, evidence from the literature suggests that these proteins could be involved in membrane trafficking and the rearrangement of microtubules (Huesmann et al. 2012). For instance, ERK1 mutants exhibit increased trichome branch numbers (Reiner et al. 2015), likely due to incorrect microtubule rearrangements (Mathur et al. 1999). Similarly, a transient knockdown of the barley *RBK1* homolog, *HvRBK1*, leads to defects in cortical microtubule stability in epidermal cells (Huesmann et al. 2012). Indeed, microtubules have also been shown to play an important role in the polar deposition of subcellular components and proteins that are involved in cell wall formation (Marchant 1979). EXO70A1, which is important for the polar localization of CASP proteins at the site of CS formation, together with other members of the exocyst complex, is also present at the site of secondary cell wall thickenings in tracheary elements, in a microtubule-dependent manner (Kalmbach et al. 2017, Vukasinovic et al. 2017).

In addition, we identified TOL6 and TIC, two significant downstream components of the ERK1-mediated signaling pathway for CS formation. TOL6 is part of the plant Endosomal Sorting Complex Required for Transport (ESCRT) complex (Moulinier-Anzola et al. 2014, Sauer and Friml 2014) and a member of a gene family that acts redundantly to control plant morphogenesis (Korbei et al. 2013). In our study, *tol6* mutants did not show obvious CS apoplastic barrier defects; however, the quintuple mutant (*tol2-1/tol3-1/tol5-1/tol6-1/tol9-1*; *tolQ*) exhibited strong ectopic lignin deposition similar to *erk1-3/rbk1-1* mutants. The ectopic lignification observed in *tolQ* did not appear to interfere with apoplastic barrier function, suggesting that lignin deposition in this mutant is probably a secondary effect of the disruption to vesicle transport. This idea is supported by the fact that together with microtubules, membrane vesicle transport plays a critical role in the localization of plant cell wall components (McFarlane et al. 2013, Miao and Liu 2010). TIC is one of the main regulators of the circadian clock (Davis and Millar 2001, Sanchez-Villarreal et al. 2013) and has been shown to be an integral component of iron homeostasis in roots (Duc et al. 2009, Sanchez-Villarreal et al. 2013). Our analysis revealed that although *tic1-2* mutants show apoplastic barrier defects and ectopic deposition of lignin in the CS, this did not interfere with the polar localization of CASP1 in the endodermis. TIC is a major regulator of the circadian clock (Davis and Millar 2001, Sanchez-Villarreal et al. 2013); yet, the CS function defects observed in *tic-2* are not a direct cause of circadian rhythm defects. TIC has also been implicated in metabolic homeostasis, particularly in sugar production or its responses (Sanchez-Villarreal et al. 2013), both of which are critical factors in cell wall formation. The striking resistance to drought observed in *tic1*-2 mutants (Sanchez-Villarreal et al. 2013) could be explained by an enhanced suberization of the endodermis that has also been shown in other CS mutants (Baxter et al. 2009). Our analysis has revealed that TIC plays an important role in the development of the root endodermis and that TIC function is likely regulated by phosphorylation. Indeed, *TIC* has also been found to interact genetically with *SNF1 KINASE HOMOLOG 10 (AKIN10)/SNF1-RELATED PROTEIN KINASE 1.1 (SnRK1.1)* to regulate circadian periodicity (Shin et al. 2017). Collectively, these data indicate that the ERK1-TIC module provides a molecular link between metabolic homeostasis and nutrient uptake ability in plants.

Plant roots secrete sugars and other metabolites into the soil as a means of attracting beneficial microbes and defending themselves against pathogens, which ultimately shapes the microbial communities present in the rhizosphere (Lanoue et al. 2010, Sasse et al. 2018). Root exudates are highly variable and defined by many parameters including plant accession (Monchgesang et al. 2016a, 2016b), developmental stage, abiotic stresses (Carvalhais et al. 2013, Chaparro et al. 2014), and more recently by the circadian clock (Hubbard et al. 2018). Given that the CS and suberin lamellae are regulators of water and nutrient uptake in the root endodermis, it is plausible that perturbations in these structures could also shape the composition of the root microbiome by altering exudate secretion, as observed in this study. Indeed, we showed that *erk1-3* and *tic-2* recruit similar microbial populations to their rhizosphere that differ markedly from the wild-type. Alternatively, modifications in the rhizosphere microbiome could be caused by differences in actively secreted compounds associated with suberization because our analysis has revealed that mutants defective in this pathway display similarities in the production of root exudates. Suberin lamellae are not thought to affect the apoplastic barrier directly but instead modulate transport through the endodermis (Andersen et al. 2015, Robbins et al. 2014), while some suberin components display antimicrobial properties (Buskila et al. 2011, Lulai and Corsini 1998, Ranathunge et al. 2008, Thomas et al. 2007). Thus, the increased suberization observed in *erk1-3* and *tic-2* could account for the specific microbial communities present in these mutant roots (Fig.�5).

**Fig. 5:**
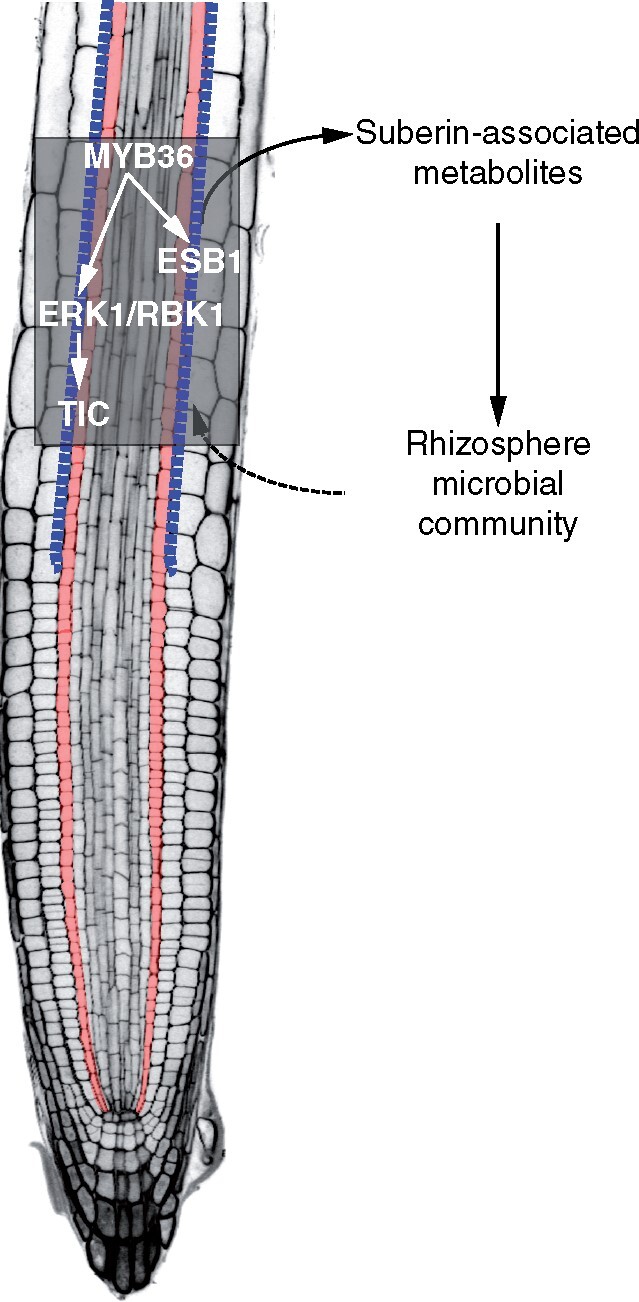
Hypothetical model for the role of the endodermal suberin pathway in rhizosphere microbial composition. The transcription factor MYB36 activates *ESB1* and *ERK1* in the endodermis. Activation of the ERK1/RBK1 signaling cascade modulates TIC activity and influences suberin deposition at the CS. Suberin-associated metabolites are actively exudated and shape the microbial community composition of the rhizosphere. Blue dotted line, suberin deposition at CS; Orange cells, endodermis.

Taken together, our work provides evidence for a complex signaling cascade taking place at the root endodermis that is necessary for both the formation of a functional CS diffusion barrier and for correct accumulation of suberin and secretion of metabolites, which ultimately determines the microbial composition of the rhizosphere.

## Materials and Methods

### Plant lines and growth conditions

All plant material used in this study was derived from the wild-type Columbia (Col-0) or Wassilewskija (Ws) accession. T-DNA insertion alleles of *TIC* (*tic-2*, SAIL_753_E03 and *tic-3*, SAIL_60_D02), *ERK1* (*erk1-1*, *rlck vi_a3-1*: SALK_148741; *erk1-2*, *rlck vi_a3-2*: SALK_010841 and *erk1-3*, *rlck vi_a3-3*: SALK_060966) and *RBK1* (*rbk1-1*: SALK_043441) were obtained from the Nottingham Arabidopsis Stock Centre (NASC). Mutant and wild-type (Col-0 or Ws) seeds were sown on soil (John Innes and Perlite mix), stratified for 2 d at 4�C in the dark and grown at 20�C under long photoperiod conditions (150 �mol/m2/s and 16 h/8 h light/dark cycles) to induce flowering. Seeds were surface sterilized for 3 min in 70% ethanol, followed by a 2-min treatment in 1% NaOCl. Seeds were then washed six times in sterile H_2_O, dispersed in sterile 0.1% agarose and placed on half-strength MS medium (Murashige & Skoog, Sigma), solidified with 0.8% Phytagar. Seedlings were grown vertically in growth chambers at 22�C, under long days (16-hr light/8-hr dark), 100 μE light, and were used at 5 d after shift to room temperature. For the evaluation of growth effect of excess iron, plants were grown on medium containing 5 mM KNO_3_, 2.5 mM KPO_4_, 3 mM MgSO_4_, 3 mM Ca(NO_3_)_2_, 70 �M H_3_BO_3_, 14 �M MnCl_2_, 0.5 �M CuSO_4_, 1 �M ZnSO_4_, 0.2 �M Na_2_MoO_4_, 10 �M NaCl, 0.01 �M CoCl_2_ and 50 �M, 300 �M or 500 �M Fe-EDTA, solidified with 0.8% phytoagar. Around 30 plants per line were grown on each plate. After 14 d, the fresh weight of all plants of one line per plate was measured. Relative fresh weight was calculated by normalization of the iron excess samples (300 �M and 500 �M Fe-EDTA) by the mock control (50 �M Fe-EDTA).

### Vector construction and plant transformation

For generation of expression constructs, Gateway Cloning Technology (Invitrogen) was used. pERK1::ERK1 was cloned using the genomic sequence of ERK1 including the endogenous promoter consisting of 1403 bp upstream of the ATG. pERK1::ERK1 was cloned into pGWB440 and into pFAST-R01 and transformed in Col-0 and *erk1-3* background, respectively. pRBK1::RBK1 was cloned using the genomic sequence of RBK1 including the endogenous promoter consisting of 1996 bp upstream of the ATG. pRBK1::RBK1 was cloned into pGWB440, pGWB553 and into pFAST-R07 and transformed in in Col-0 background. Transgenic plants were generated by introduction of the plant expression constructs into an *Agrobacterium tumefaciens* strain GV3101 and transformation was done by floral dipping (Clough and Bent 1998).

### Chromatin immunoprecipitation

Chromatin immunoprecipitation (ChIP) analysis was performed by following the protocol as described (Nakamichi et al. 2010) with modifications. Roots (100 mg fresh weight) from 11d-old plants were cross-linked by using 4 ml of the buffer (10 mM PBS, pH 7.0, 50 mM NaCl, 0.1 M sucrose and 1% formaldehyde) for 1 h at room temperature with the application of three cycles of vacuum infiltration (10 min under vacuum and 10 min of vacuum release). Glycine was added to a final concentration of 0.1 M to stop the cross-linking reaction, and the samples were incubated for a further 10 min. After being washed with tap water, the samples were ground to a fine powder by using a Multibeads Shocker (Yasui Kikai) at 1,500 rpm for 30 sec. The powder was suspended with 2 ml of Lysis buffer [50 mM Tris�HCl, pH 7.5, 100 mM NaCl, 1% Triton X-100, 1 mM EDTA and EDTA-free Complete protease inhibitor (Roche)] and sonicated by using a Bioruptor UCD-250 (Cosmo Bio) with the following setting: mild intensity, 45 cycles (30 sec ON and 30 sec OFF) at 4�C. A 100-μL sample of the chromatin sheared to between 200 and 1,500 bp was stored as the input fraction, and the rest (1.9 ml) was mixed with Dynabeads Protein G (Life Technologies) bound with anti-GFP antibody (ab290; Abcam) and incubated for 2 h at 4�C. The beads were washed with Lysis buffer, twice with high-salt buffer [50 mM Tris�HCl, pH 7.5, 400 mM NaCl, 1% Triton X-100, 1 mM EDTA and EDTA-free Complete protease inhibitor (Roche)], and then with Lysis buffer. After Elution buffer (50 mM Tris�HCl, pH 8.0, 10 mM EDTA and 1% SDS) and proteinase K (0.5 mg/ml) were added to the beads, the beads were incubated overnight at 65�C. The DNA was purified with NucleoSpin Gel and PCR Clean-up (MachereyNagel) with Buffer NTB (Macherey-Nagel). Eluted solutions were used for qPCR. EIF4A (At3g13920) was used as a negative control (Supplementary Table S2). Two independent experiments were performed with three biological replicates for each.

### Permeability of the apoplastic barrier

For the visualization of the penetration of the apoplastic barrier by propidium iodide, seedlings were incubated in the dark for 10 min in a fresh solution of 15 mM (10 mg/ml) propidium IIodide (PI) and rinsed two times in water. The penetration of the apoplastic barrier was quantified by the number of cells from the ‘onset of elongation’ until the PI signal was blocked by the endodermis from entering the vasculature. The ‘onset of elongation’ was defined as the point where an endodermal cell in a median optical section was clearly more than twice its width (Alassimone et al. 2010).

### Histological analysis

Tissue was fixed and stained as described previously (Musielak et al. 2016). In brief, 5-day-old seedlings were fixed in 4% paraformaldehyde (PFA) and cell walls were stained with SCRI Renaissance 2200 (SR2200) (0.1% (v/v) SR2200, 1% (v/v) DMSO, 0.05% (v/v) Triton-X 100, 5% (v/v) glycerol and 4% (w/v) paraformaldehyde in PBS buffer (pH 7.4)) in PBS for 15 min at room temperature with no vacuum applied. Fixed seedlings were washed twice with PBS (pH 7.4) and cleared with ClearSee (10% xylitol (w/v), 15% sodium deoxycholate (w/v) and 25% urea (w/v) in water) for 4 d at room temperature (Kurihara et al. 2015). Cleared samples were washed twice with PBS (pH 7.4) and embedded in 4% agarose. The agarose blocks were cut into 200 �m sections with a VIBRATOME^�^ Series 1000 Sectioning System.

### Electron microscopy analysis

For light transmission electron microscopy (TEM), roots were dehydrated in an ethanol/propylene oxide series, embedded in Spurr’s resin (Premix Kit-Hard, TAAB Laboratory and Microscopy, Aldermaston, UK) and polymerized at 70�C overnight. Longitudinal ultrathin sections (60 nm) were cut using an ultramicrotome (ultra-RMC Products), mounted on copper grids and contrasted with 1 % uranyl acetate in water for 25 min followed by lead citrate for 3 min. Sections were examined in a transmission electron microscope (Spirit Biotwin 12 FEI Company). Measurements were carried out using the TEM Imaging Platform program.

### Confocal microscopy

Confocal laser scanning microscopy experiments were performed using a Zeiss Axio Observer.Z1 inverted microscope equipped with a confocal laser-scanning module (Zeiss LSM 710 and Zeiss LSM 880, Warwick) or a Leica SP5 and SP8 (Nottingham).

Excitation and emission setting were used as follows: GFP 488 nm, 500–550 nm; propidium iodide 516 nm, 560–700 nm; tagRFP 561 nm, 578–700 nm; SR2200 405 nm, 410–550 nm; calcofluor white 405 nm, 425–475 nm; and basic fuchsin 561 nm, 570–650 nm. For examining CASP1 expression, basic fuchsin and calcofluor white M2R staining, 6d-old roots were fixed in paraformaldehyde and cleared in ClearSee as described (Ursache et al. 2018). Fluorol yellow staining for visualization of suberin was performed and quantified as described in Barberon et al. (2016) and Naseer et al. (2012), using a fluorescent microscope Leica DM 5000. Confocal images were analyzed and contrast and brightness were adjusted with the Fiji package of ImageJ (http://fiji.sc/Fiji) and Adobe Photoshop Elements Editor.

The confocal images of the pERK1::ERK1-GFP reporter under different iron concentrations were taken with a Zeiss Axio Observer.Z1 inverted microscope equipped with a confocal laser-scanning module (Zeiss LSM 880). The exact same settings were used during one experiment. To be able to compare different roots, optical median sections were made. The florescent area was selected with FIJI (ImageJ) and the area, area-integrated intensity and mean gray value of each image were measured. Intensities were corrected with the mean intensity of areas without signal (background). The corrected total fluorescence (CTF) was calculated by using this formula: CTF=integrated density−(selected fluorescent area�mean background fluorescence).

### Phosphoproteomic analysis

For the purification of total proteins, root seedlings were grown hydroponically in phytatrays (Sigma) on a nylon filter (250 μm mesh; NITEX) which allows the roots to grow through into the half-strength MS medium supplemented with 1% (w/v) sucrose (pH 5.7 with KOH) (Bargmann and Birnbaum 2010). After 2 weeks, roots were harvested and flash-frozen in liquid nitrogen. The root tissue was homogenized with a mortar and pestle cooled with liquid nitrogen. Proteins were extracted by adding twice the volume of extraction buffer (50 mM HEPES, 150 mM NaCl, 1 mM EDTA, 20 mM NaF, 1 mM Na2MoO4, 1% (w/v) NP-40, 1 mM PMSF, 2 �M Calyculin A, 1 mM NaVO4, 1 mM DTT, Protease inhibitor cocktail (Roche) and 2% (w/v) PVPP) to 3 g of root tissue. After 30 min, the samples were spun for 15 min at 4,000 g (4�C) to remove debris. The supernatant was transferred to a new tube and centrifuged for another 30 min at 16,000 � *g* (4�C). The supernatant was again transferred to a new tube and methanol/chloroform precipitation was carried out by adding 4 volumes of methanol, 1 volume of chloroform and 3 volumes of water, respectively. Samples were centrifuges for 15 min at 4,000 � *g* and the aqueous top phase was removed. The proteins were precipitated by adding another 4 volumes of methanol and centrifugation for 15 min at 4,000 � *g*. The pellet was washed twice with methanol and resuspended in 25 mM HEPES (pH 8). Reduction and alkylation of the cysteine residues were carried out by adding a combination of 5 mM tris(2-carboxyethyl)phosphine (TCEP) and 10 mM iodoacetamide (IAA) for 30 min at room temperature in the dark. The protein was digested with trypsin (Promega Trypsin Gold, mass spectrometry grade) overnight at 37�C at an enzyme-to-substrate ratio of 1:100 (w: w). After the digestion, the peptides were suspended in 80% acetonitrile (AcN), 5% trifluoroacetic acid (TFA) and the insoluble matter was spun down at 4,000 g for 10 min. The supernatant was used the enrichment of phosphopeptides.

The enrichment of phosphopeptides was carried out as described previously with minor modifications (Thingholm et al. 2006). The peptide concentration was measured with a Qubit^™^ fluorometer (Invitrogen) and 1 �g of total peptides was used for each sample. The Titansphere TiO_2_ 10 �m beads (GL Sciences Inc.) were equilibrated in a buffer containing 20 mg/ml 2,5-dihydroxybenzoic acid (DHB), 80% ACN and 5% TFA in a ratio of 10 �l DHBeq per 1 mg beads for 10 min with gentle shaking at 600 rpm. TiO2 beads were used in a ratio of 1:2 peptide-bead ration (w: w). The TiO2 solution was added to each sample and incubated for 60 min at room temperature. The samples were spun down at 3,000 g for 2 min and resuspended in 100 �l Wash buffer I (10% AcN, 5% TFA). The resuspended beads were added to self-made C8-columns. C8-colums were made of 200 �l pipette tips with 2 mm Empore™Octyl C8 (Supelco) discs. The columns were spun down at 2,600 g for 2 min, washed with 100 �l Wash buffer II (40% AcN, 5% TFA) and 100 �l Wash buffer III (40% AcN, 5% TFA). The peptides were eluted from the TiO_2_ beads with 20 �l 5% ammonium hydroxide and subsequently with 20 �l 20% ammonium hydroxide in 25% AcN. Both eluates were pooled, the volume was reduced to 5 �l in a centrifugal evaporator (20–30 min) and acidified with 100 �l of buffer A (2% AcN, 1% TFA). Samples were desalted with a self-made C18 column (Empore™Octadecyl C18). C18 were made in the same way as the C8-columns. Before adding the samples, the C18-columns were activated with 50 �l methanol and washed with 50 �l AcN and 50 �l buffer A* (2% AcN, 0.1% TFA). Samples were loaded on the C18-column and spun at 2,000 g for 7 min. The columns were washed with 50 �l ethyl acetate and 50 �l buffer A* and then eluted consecutively with 20 �l 40% AcN and 20 �l 60% AcN. Samples were then vacuum-dried and prior to MS analysis resuspended in 50 �l buffer A*.

### Mass spectrometry

Reversed phase chromatography was used to separate tryptic peptides prior to mass spectrometric analysis. Two columns were utilized, an Acclaim PepMap �-precolumn cartridge 300 �m i.d. x 5 mm 5 μm 100 � and an Acclaim PepMap RSLC 75 �m x 25 cm 2 �m 100 � (Thermo Scientific). The columns were installed on an Ultimate 3000 RSLCnano system (Dionex). Mobile phase buffer A was composed of 0.1% formic acid in water and mobile phase B 0.1 % formic acid in acetonitrile. Samples were loaded onto the �-precolumn equilibrated in 2% aqueous acetonitrile containing 0.1% trifluoroacetic acid for 8 min at 10 �l min-1 after which peptides were eluted onto the analytical column at 300 nL min-1 by increasing the mobile phase B concentration from 3% B to 35% over 40 min and then to 90% B over 4 min, followed by a 15-min re-equilibration at 3% B.

Eluting peptides were converted to gas-phase ions by means of electrospray ionization and analyzed on a Thermo Orbitrap Fusion (Q-OT-qIT, Thermo Scientific). Survey scans of peptide precursors from 350 to 1,500 m/z were performed at 120 K resolution (at 200 m/z) with a 4 � 105 ion count target. Tandem MS was performed by isolation at 1.6 Th using the quadrupole, HCD fragmentation with normalized collision energy of 35, and rapid scan MS analysis in the ion trap. The MS2 ion count target was set to 1x104 and the max injection time was 200 ms. Precursors with charge state 2–7 were selected and sampled for MS2. The dynamic exclusion duration was set to 45 sec with a 10 ppm tolerance around the selected precursor and its isotopes. Monoisotopic precursor selection was turned on. The instrument was run in top speed mode with 2 sec cycles.

### Phosphoproteomic data analysis

A label-free peptide relative quantification analysis was performed in Progenesis QI for Proteomics (Nonlinear Dynamics, Durham). To identify peptides, peak lists were created by using Progenesis QI. The raw data were searched against Arabidopsis TAIR database and the common Repository of Adventitious Proteins (http://www.thegpm.org/cRAP/index.html) using uninterpreted MS/MS ions searches within Mascot engine (http://www.matrixscience.com/). Peptides were generated from a tryptic digestion with up to two missed cleavages, carbamidomethylation of cysteines as fixed modifications, oxidation of methionine and phosphorylation of serine, threonine and tyrosine as variable modifications. Precursor mass tolerance was 5 ppm and product ions were searched at 0.8 Da tolerances.

Scaffold (TM, version 4.4.5, Proteome Software Inc.) was used to validate MS/MS-based peptide and protein identifications. Peptide identifications were accepted if they could be established at greater than 95.0% probability by the Scaffold Local FDR algorithm. Protein identifications were accepted if they could be established at greater than 99.0% probability and contained at least one identified peptide. Proteins that contained similar peptides and could not be differentiated based on MS/MS analysis alone were grouped to satisfy the principles of parsimony.

### Ionome analysis

Ionomic analysis of plants grown liquid media agar was performed as described in Hosmani et al. (2013). Plants were grown on liquid medium (0.25 mM CaCl_2_ 2 H_2_O, 1 mM KH_2_PO_4_, 0.05 mM KCl, 0.25 K_2_SO_4_, 1 mM MgSO_4_ 7 H_2_O, 0.1 mM NaFe-EDTA, 2 mM NH_4_NO_3_, 30 �M H_3_BO_3_, 5 �M MnSO_4_ 5 H_2_O, 1 �M ZnSO_4_ 7 H_2_O, 1 �M CuSO_4_ 5 H_2_O, 0.7 �M NaMoO_4_ 2 H_2_O and 1 �M NiSO_4_ 6 H_2_O) in short day condition (10 h light 20�C/14h dark 18�C). Six plants were grown in boxes containing 500 ml of media. The media was renewed weekly. After 5 weeks, shoots were harvested, dried at 88�C for 20 h and digested with concentrated nitric acid. Digested samples were diluted with 18 MΩ water and analyzed by using inductively coupled plasma (ICP)-MS (NexION 2000; PerkinElmer).

### Natural soil microbiome

For the root microbiome analysis on natural soil, we used sandy loam (pH 6.7, 1.4% organic carbon) from the University of Warwick Crop Centre (Wellesbourne, UK). Seeds were sown in 1.5 in x 1.5 in pots and stratified for 2 d at 4�C. Seeds from the different lines were sown using a randomized scheme and plants were grown under controlled environment conditions (12 h light/12h dark, 22�C) for 4 weeks. DNA was extracted from rhizosphere samples (root and adhering soil) using the Griffith method (Griffith et al. 2000). DNA was normalized to 5 ng/μl before PCR amplification. For each sample, 15 ng of DNA was used to amplify part of the 16S rRNA gene using primer pairs 515f and 806r (Caporaso et al. 2011). The primer set was modified at the 5′ end with Illumina Nextera Index Kit v2 adapters. PCR reactions were performed in a reaction volume of 25 μl, containing Q5^�^ Hot Start High-Fidelity 2X Master Mix (New England Biolabs) and 0.5 �M of each primer. Thermocycling consisted of an initial denaturation at 98�C for 30 s followed by 25 cycles of 98�C for 10 s, 50�C for 15 s and 72�C for 20 s with a final extension at 72�C for 5 min. Following PCR, DNA amplicons were purified using Agencourt AMPure XP beads (Beckman Coulter, USA) according to the manufacturer’s instructions. The adapted amplicons were then modified by attaching indices and Illumina sequencing adapters using the Nextera XT Index Kit v2 by PCR as described in the manufacturer’s protocol, enabling simultaneous sequencing of multiple samples. Following the index PCR, the DNA amplicons were purified and normalized using the SequalPrep™ Normalization Plate (96) Kit (Invitrogen) and then quantitatively assessed using a Qubit 2.0 Fluorometer (Life Technologies, USA). The final concentration of the pooled library was 4 nM. The library was sequenced using the MiSeq Reagent Kit v3 600-cycle (Illumina).

Following sequencing, Trimmomatic v0.35 was used to remove low-quality bases from the sequence ends (Bolger et al. 2014). The following steps were then performed using USEARCH and UPARSE software (Edgar 2010, 2013). Paired-end reads were assembled by aligning the forward and reverse reads, trimming primers and quality filtering (-fastq_maxee 0.5). Unique sequences were sorted by abundance and singletons were discarded from the dataset. Sequences were clustered to OTUs at 97% minimum identity threshold using -cluster_otus, which includes chimera filtering. This was followed by further chimera filtering using the gold database (Edgar et al. 2011). Sequences were clustered to OTUs at 97% minimum identity threshold. Taxonomy was assigned using Quantitative Insights into Microbial Ecology (QIIME 1.8) and the Greengenes reference database (Caporaso et al. 2010, McDonald et al. 2012). Mitochondrial and chloroplast sequences were removed from the dataset. A total 896,362 sequence reads were assigned to bacteria and used in subsequent analyses with an average of 12,805 reads per sample.

The Phyloseq package (version 1.6.0) in the R software environment was applied for data interpretation and graphical representation including nonmetric multidimensional scaling (NMDS) (McMurdie and Holmes 2013).

### SynCom microbiome analysis

The effect of host genotype on plant-associated bacterial community structure was tested using recolonization experiments with a gnotobiotic system based on calcined clay inoculated with a SynCom of 206 At-RSPHERE root isolates complemented with 30 soil abundant isolates (Bai et al. 2015). In brief, 100 g of dry sterile calcined clay in Magenta boxes was mixed with 70 ml � MS medium containing carrier solution (10 mM MgSO_4_) and/or the bacterial SynCom, resulting in about 2.75*106 cells per g calcined clay.

One-week-old seedlings were transferred from germination plates to the Magenta boxes at 4 seedlings per box and further incubated for 7 weeks. For each plant genotype, three independently prepared SynComs were inoculated in three technical replicates. At harvest, rosette, roots and clay of unplanted boxes were harvested and DNA was isolated (including the input SynComs) using the FastDNA kit for Soil after milling harvested materials in a 2-ml lysing matrix E tube (MP Biomedicals). To assess the bacterial community structure, a 16S rRNA gene-based analysis was employed. The variable regions V5-V7 of bacterial 16S rRNA genes were amplified by PCR. In a second PCR, Illumina adapters and barcodes were added to products of the first PCR. All samples were gel purified and pooled in equimolar amounts and the final amplicon libraries were twice purified using Agencourt AMPure XP beads (Beckman Coulter) and sequenced on the Illumina MiSeq platform using the MiSeq Reagent kit v3 (2� 300 bp pair-end reads and 12 bp barcode).

Forward and reverse reads were joined, demultiplexed and subjected to quality control using a combination of QIIME and USEARCH pipelines (Caporaso et al. 2010, Edgar 2010). High-quality sequences were clustered to 99% sequence identity together with reference sequences of root and soil isolates assembled in the SynCom using the UPARSE algorithm (Edgar 2013). OTUs corresponding to one or more reference 16S rRNA gene sequence(s) were selected and an OTU table was generated with read frequencies. Beta-diversity measures (Bray-Curtis) were calculated and canonical analysis of principle coordinates was computed on the distance matrix with the capscale function implemented in the vegan R package. Significance of the constrained variable ‘genotype’ was calculated by a permutation-based ANOVA test using 1,000 permutation. Additionally, relative abundances of the 10 most abundant families (based on the average family abundance across all samples) were calculated and visualized as stacked barplots using ggplot2. Analysis of variance with Student-Newman-Keuls, as post-hoc test as implemented in the agricolae R package, was used to assess significant differences in relative abundance.

### Metabolomic analysis of root exudates

Plants were grown for 2 weeks in phytatrays containing a � MS nutrient solution. Before collection, the media was tested for bacterial contamination by inoculation on LB plates. Roots were washed twice with sterile deionized water and transferred to phytatrays containing sterile deionized water. Two days later, water containing root exudates was collected, freeze dried and subjected to liquid chromatography-mass spectrometry (LC-MS) analysis.

## Funding

This work was supported by JSPS KAKENHI grant (17H03782) to T.K., ERC AdG ROOTMICROBIOTA, CEPLAS and Max Planck Society to P.S.L., and BBSRC grants (BB/N023927/1 and BB/L027739/1) to D.S. and (BB/L025892/1) to G.D.B. and (BB/L003023/1, BB/N005279/1, BB/N00194X/1 and BB/P02601X/1) to J.G.-M.

## Supplementary Data

Supplementary data are available at PCP online.

## Supplementary Material

pcaa170_Supplementary_Data

## Data Availability

The mass spectrometry proteomics data have been deposited to the ProteomeXchange Consortium via the PRIDE partner repository with the dataset identifier PXD013861 and 10.6019/PXD013861. Sequencing reads have been deposited at the NCBI SRA Archive (accession no. PRJNA543397).
