# Supplementary material for: A Novel Signaling Pathway Required for Arabidopsis Endodermal Root Organization Shapes the Rhizosphere Microbiome
Source: Plant Cell Physiol. 2021 Jan 22;62(2):248–61. doi: 10.1093/pcp/pcaa170 (PMC8112839; doi:10.1093/pcp/pcaa170)
Supplement: pcaa170_Supplementary_Data [file pcaa170_supplementary_data.zip › Durr_et_al._Suppl_Info.docx]

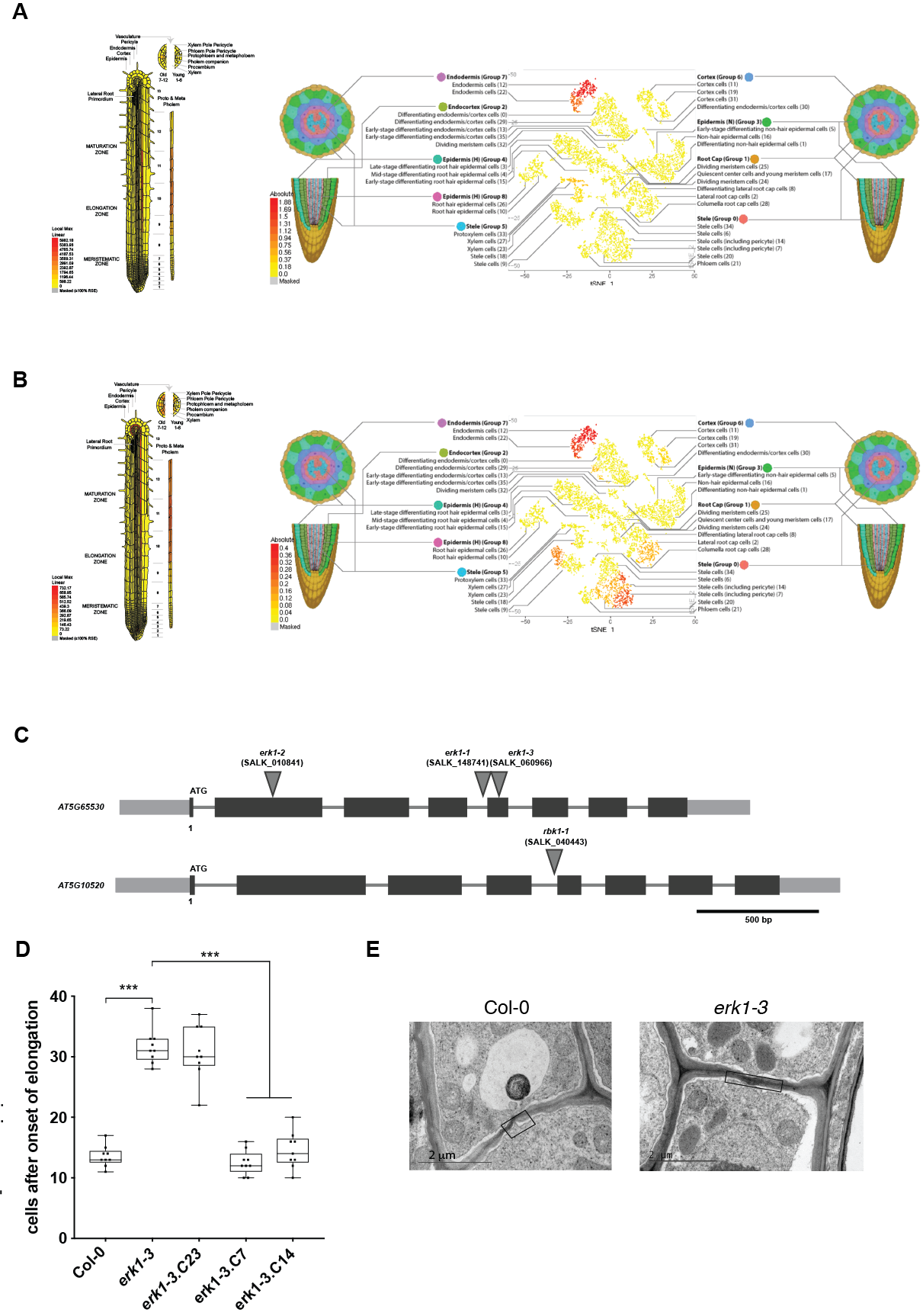


**Supplementary figure 1. *ERK1* and *RBK1* are expressed in the root endodermis and are implicated in CS function.**

(A) *ERK1* (AT5G65530) is specifically expressed in root endodermis cells. eFP Browser views of Arabidopsis root expression (Brady et al., 2007) (Left) and single cell RNA-seq from roots (Ryu et al., 2019) (Right).

(B) *RBK1* (AT5G10520) is specifically expressed in root stele and endodermis cells. eFP Browser views of Arabidopsis root expression (Brady et al., 2007) (Left) and single cell RNA-seq from roots (Ryu et al., 2019) (Right).

(C) Isolation of independent mutant alleles for *ERK1* and *RBK1­*.

(D) Functional complementation of *erk1-3* with pERK1-ERK1. Three complementation lines (C7, C14 and C23) were randomly selected for quantification of PI penetration into the stele, as number of endodermal cells from the first fully expanded cell (n = 15). Differences between groups were determined by paired t-test, ***p < 0.001.

(E) Transmission Electron Microscopy images showing the CS cell wall deposition in the endodermis of Col-0 and *erk1-3* plants. Scale bar, 2 μm. Black box, cell wall deposition at CS.

**Supplementary figure 2. Quantitative phosphoproteomic analysis in *erk1-3* roots identified TIC and TOL6 as downstream targets.**

(A) Gene ontology analysis of proteins differentially phosphorylated in *erk1-3* roots (> 1.5-fold change, p-value 0.001).

(B) Serine residues differentially phosphorylated in TIC and TOL6 between Col-0 and *erk1-3* (n=2). Differences between groups were determined by paired t-test, **p < 0.001.

**Supplementary figure 3. Apoplastic barrier integrity in TOL and circadian clock mutants.**

(A) Quantification of PI diffusion into the stele in different *tol* mutant combinations. PI diffusion was quantified as number of endodermal cells from the first fully expanded cell (n = 10). Differences between groups were determined by paired t-test; n.s., no significant.

(B) Quantification of PI diffusion into the stele quantified in mutant defective in circadian rhythm regulation. PI diffusion was quantified as number of endodermal cells from the first fully expanded cell (n = 10). Differences between groups were determined by paired t-test; ***p<0.001, n.s., no significant.


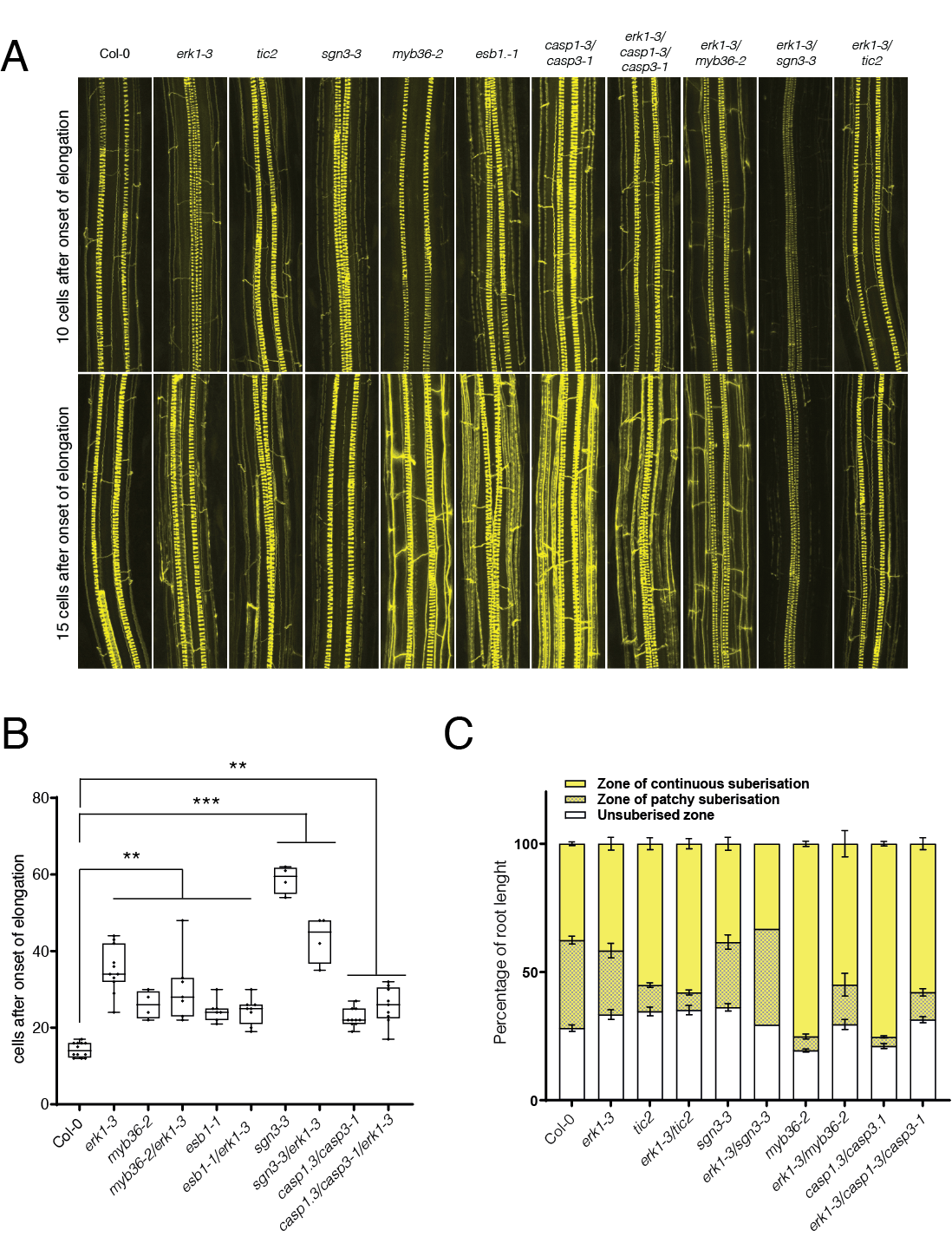


**Supplementary figure 4. Analysis Casparian strip integrity in different mutants.**

(A) Lignin deposition in different casparian strip mutants and different mutant combinations.

(B) Quantification of PI penetration into the stele of endodermal cells of casparian strip mutants and different mutant combinations. Differences between groups were determined by paired t-test; ***p<0.001, **p<0.01.

(C) Quantification of suberinisation of endodermal cells in different casparian strip mutants (n=6). Individual letter shows significant differences using Mann-Whitney test between the same zones (p<0.01).


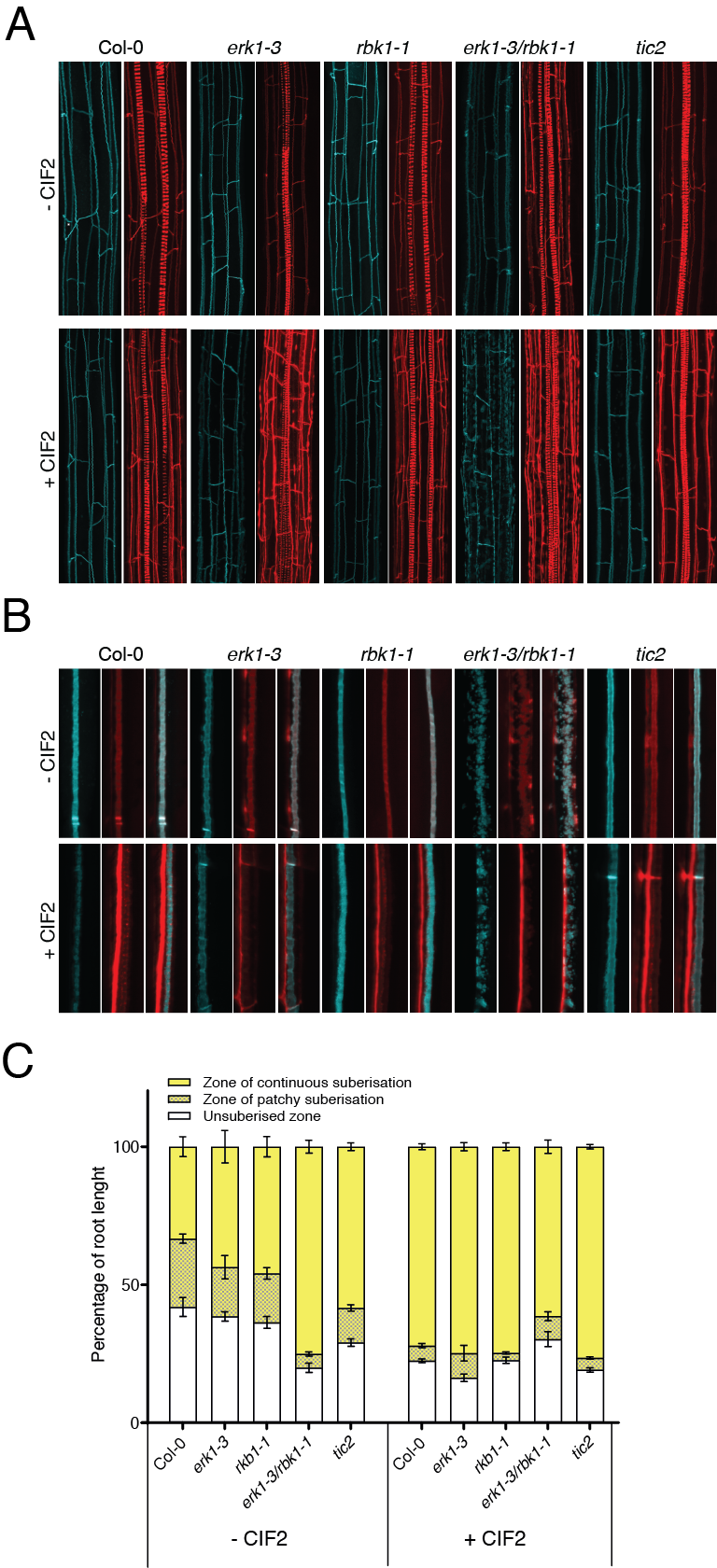


**Supplementary figure 5. Distribution of lignin and CASP1-GFP in roots of Casparian strip mutants upon exposure to CIF2 peptides.**

(A) ﻿ Lignin deposition in root endodermis after exposure to CIF2 peptides. Red fluorescence, lignin; Blue fluorescence, cell wall.

(B) CASP1-GFP deposition in root endodermis after exposure to CIF2 peptides. Red fluorescence, basic Fuchsin; Blue fluorescence, CASP1-GFP.

(C) Distribution of suberin in roots of Casparian strip mutants upon exposure to CIF2 peptides (n=6). Individual letter shows significant differences using Mann-Whitney test between the same zones (p<0.01).

**Supplementary figure 6. Inonome analysis and effect of excess iron on plant growth.**

(A) ﻿Principal component analysis based on the inomic concentration of 16 elements in shoots (n=6).

(B) Effect of excess iron in vegetative growth of different Casparian strip mutants (n=10). Differences between groups were determined by paired t-test, ***p < 0.001.


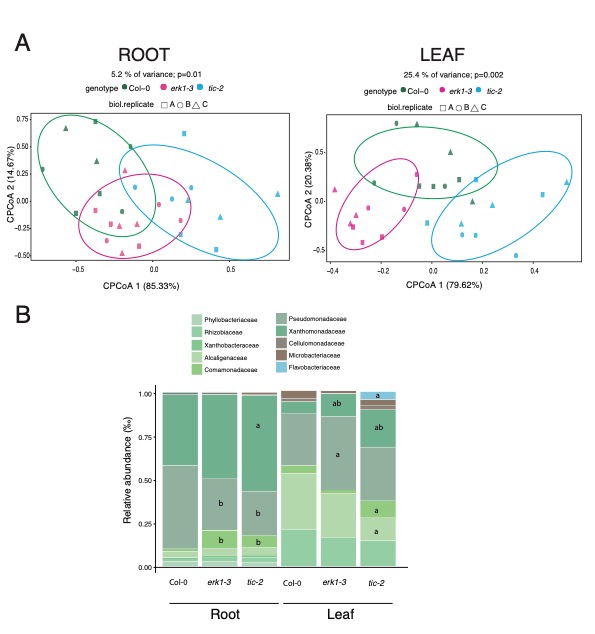


**Supplementary figure 7. Bacterial communities of CS mutants inoculated with SynCom cultured communities.**

﻿(A) Canonical analysis of principal coordinates (based on Bray–Curtis distances) showing different root- and leaf-associated communities of SynComs (n = 10).

(B) Relative abundances of the 10 most abundant bacterial families. Individual letter shows significant differences using Mann-Whitney test between the same zones (p > 0.01).­­
