# Supplementary figures and images for: A Novel Signaling Pathway Required for Arabidopsis Endodermal Root Organization Shapes the Rhizosphere Microbiome
Source: Plant Cell Physiol. 2021 Jan 22;62(2):248–61. doi: 10.1093/pcp/pcaa170 (PMC8112839; doi:10.1093/pcp/pcaa170)

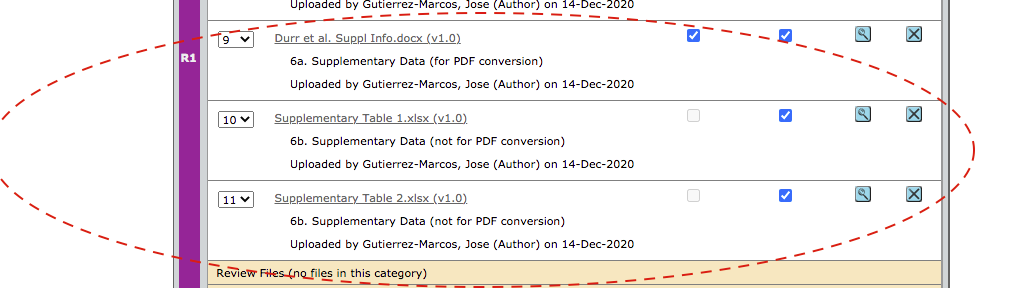

Supplement: pcaa170_Supplementary_Data [file pcaa170_supplementary_data.zip › Screen Shot 2021-01-21 at 12.48.40.png]
